# Mass loss and chemical structures of wheat and maize straws in response to ultraviolet-B radiation and soil contact

**DOI:** 10.1038/srep14851

**Published:** 2015-10-01

**Authors:** Guixiang Zhou, Jiabao Zhang, Jingdong Mao, Congzhi Zhang, Lin Chen, Xiuli Xin, Bingzi Zhao

**Affiliations:** 1 State Key Laboratory of Soil and Sustainable Agriculture, Institute of Soil Science, Chinese Academy of Sciences, Nanjing 210008, China; 2 Jiujiang University, Jiujiang 332005, China; 3 University of Chinese Academy of Sciences, Beijing 100049, China; 4 Department of Chemistry and Biochemistry, Old Dominion University, 4541 Hampton Boulevard, Norfolk, Virginia 23529, United States; 5 Institute of Soil and Water Resources and Environmental Science, College of Environmental & Resource Sciences, Zhejiang University, Hangzhou 310058, China

## Abstract

The role of photodegradation, an abiotic process, has been largely overlooked during straw decomposition in mesic ecosystems. We investigated the mass loss and chemical structures of straw decomposition in response to elevated UV-B radiation with or without soil contact over a 12-month litterbag experiment. Wheat and maize straw samples with and without soil contact were exposed to three radiation levels: a no-sunlight control, ambient solar UV-B, and artificially elevated UV-B radiation. A block control with soil contact was not included. Compared with the no-sunlight control, UV-B radiation increased the mass loss by 14–19% and the ambient radiation by 9–16% for wheat and maize straws without soil contact after 12 months. Elevated UV-B exposure decreased the decomposition rates of both wheat and maize straws when in contact with soil. Light exposure resulted in decreased O-alkyl carbons and increased alkyl carbons for both the wheat and maize straws compared with no-sunlight control. The difference in soil contact may influence the contribution of photodegradation to the overall straw decomposition process. These results indicate that we must take into account the effects of photodegradation when explaining the mechanisms of straw decomposition in mesic ecosystems.

Litter decomposition plays an important role in carbon (C) and nutrient cycling in the biosphere^1^. In terrestrial ecosystems, it is generally assumed that litter decomposition is driven solely by biotic processes^2^,^3^, while the role of abiotic processes in litter decomposition at the soil surface remains largely overlooked. However, photodegradation, which is the direct breakdown of litter by solar radiation, is generally known to contribute to litter decomposition rates and ultimately results in changes in C and nutrient cycling among the atmosphere, plants and soils^4^.

Ultraviolet-B (UV-B) irradiance is regarded as responsible for the vast majority of photodegradation^5^,^6^,^7^,^8^,^9^,^10^. UV-B radiation, which is comprised of high-energy photons, can induce the photochemical mineralization of plant litter^11^ and disrupt the structures of complex compounds in plant litter, especially those of lignin and polyphenols such as tannin^12^. A growing number of studies have suggested that UV-B exposure may contribute significantly to litter decomposition, resulting in increased loss of litter mass due to photodegradation^9^,^10^,^13^. Day *et al.*^14^ estimated that 14–22% of the mass loss of leaf litter can be attributed to UV-B exposure based on a photodegradation experiment carried out over 4–5 months. Through photochemical mineralization, UV-B exposure can destroy the physical structure of the litter surface, while further facilitating microbial decomposition^9^,^15^. Photochemical mineralization is the direct breakdown of litter to CO_2_ in the absence of microbial activity^11^,^16^, and microbial facilitation is the breakdown of large organic compounds by solar radiation into smaller compounds that can be utilized by microbes^5^,^14^,^17^. Elevated UV-B radiation doses often increase the mass loss of litter^18^, but not always. Some studies have indicated that UV-B exposure does not accelerate litter decomposition rates^19^, or even inhibits decomposition due to reduced microbial activity^8^.

The plant litter on the soil surface in an agroecosystem is exposed to solar radiation; as a result, the microbes within the soil can also be influenced by UV-B exposure. UV-B radiation can directly influence litter decomposition on the soil surface by changing the microbial decomposer communities^20^,^21^ and biotic processes^18^. Barners *et al.*^22^ found that the combination of litter and soil can reduce the direct effect of UV photodegradation and can ameliorate the development of soil-microbial films in the Santa Rita Experimental Range in SE Arizona, USA. This may be due to the fact that soil acts as a vector for microbial colonization, while protecting the microorganisms from high temperatures and desiccation in this ecosystem. Reports have suggested that litter placed on the soil surface may decompose faster than litter suspended aboveground without soil contact^23^,^24^. The proximity of litter to the soil surface promotes litter decomposition by offering enhanced microbial activity. The effect of UV-B exposure on litter decomposition depends on the relative contribution of photodegradation and microbial decomposition. However, it is unclear how the difference in soil contact changes the effect of UV-B radiation on straw decomposition, nor is it known how UV-B radiation drives straw degradation.

Studies associated with photodegradation have mostly been carried out in arid and semi-arid ecosystems^5^,^11^,^14^,^17^,^18^, whereas the role of photodegradation in the decomposition of litter in mesic ecosystems remains poorly understood. Previous studies of photodegradation have focused on the mass loss of litter^5^,^9^,^10^,^14^ but not on the role of photodegradation in the chemical structures within the litter. A better understanding of the chemical structures in litter during decomposition is critical for elucidating the role of photodegradation in mesic ecosystems. The primary objectives of the present study were to (1) evaluate the roles of UV-B and soil contact in decomposition of straw and (2) to examine the changes in the chemical structures of straw with and without soil contact under elevated UV-B radiation by using solid-state ^13^C NMR. We hypothesized that UV-B exposure would influence straw decomposition, with a dependence on soil contact.

## Results

### Straw mass loss

**Figure 1:**
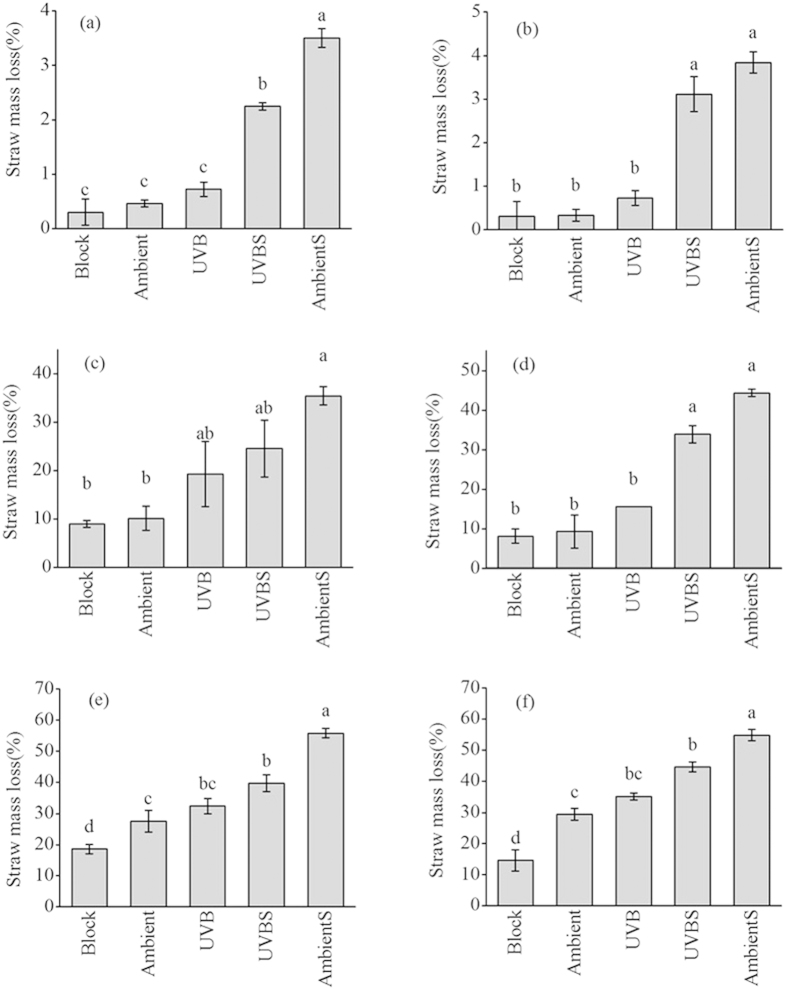
Mass loss in wheat (a,c,e) and maize (b,d,f) straws after 3 (a,b), 8 (c,d) and 12 months (e,f) of decomposition. Error bars indicate standard errors (n = 3). Lowercase letters denote significant differences between treatment groups at the 5% level according to Tukey’s test.

Figure 1 shows the straw mass loss of different treatments after 3, 8 and 12 months of decomposition. After 3 months, wheat straw lost 0.3%–3.5% of its initial mass, and maize straw lost 0.3%–3.8% of its initial mass, indicating low decomposition rates in the early stage of decomposition. In contrast, after 12 months, wheat straw lost 18.6%–55.8% of its initial mass, and maize straw, 14.6%–54.9% of its initial mass (Fig.1(e,f)). UV-B radiation had a significant effect on wheat straw mass loss (*P* = 0.005, Table 1). Compared with wheat straw without either soil contact or sunlight (Block), the mass loss of wheat straw increased by 9% (*P* < 0.05) for ambient solar radiation without soil contact (Ambient) and 14% (*P* < 0.05) for elevated UV-B exposure without soil contact (UVB) (Fig. 1e). Both UV-B radiation and soil contact had significant effects on maize straw mass loss (*P* < 0.001, Table 1). Compared with maize straw without soil contact and sunlight (Block), the mass loss of maize straw increased by 16% (*P* < 0.01) for Ambient and 19% (*P* < 0.01) for UVB (Fig. 1f). The mass losses of elevated UV-B radiation with soil contact (UVBS) for both straws were lower (*P* < 0.05) than those of ambient solar radiation with soil contact (AmbientS) (Fig. 1). The results indicated that the role of UV-B radiation in straw mass loss varied among treatments with and without soil contact (Fig. 1) and soil contact influenced straw mass loss to a highly significant degree (*P* < 0.001, Table 1). There were significant interactive effects of UV-B radiation and soil contact on wheat straw mass loss (*P* = 0.002) and maize straw mass loss (*P* = 0.026).

### Straw chemical properties

The total C and N concentrations for both the wheat and maize straws decreased as the decomposition process proceeded, except for the N concentration of the maize straw under the AmbientS condition (Table 2). For the wheat straw treatments, the straw exposed to UVB exhibited lower total C concentrations than the Ambient and Block straws, and the straw of AmbientS had the lowest total C concentration (Table 2). For the maize treatments, the straw under the Block treatment showed the highest total C concentration, whereas the C concentration of AmbientS was lower than that of Ambient (Table 2). As shown in Table 2, light exposure did not affect the N concentration of either the wheat or maize straw. As straw decomposition proceeded, the C:N ratios increased in all the treatments except the AmbientS of wheat straw and the AmbientS and UVBS of maize straw. After a 12-month decomposition period, the C:N ratios of the Block condition for both the wheat and maize straws were significantly (*P* < 0.05) higher than those of Ambient and UVB, indicating that light exposure strongly decreased the C:N ratios. The C:N ratios of AmbientS and UVBS were lower than those of Ambient and UVB for both the wheat and maize straws except for the UV-B condition of maize straw, suggesting that soil contact may regulate the C:N ratios of straw during degradation. UV-B exposure significantly (*P* < 0.05) increased the C:N ratios of both straws under soil contact, but not those without soil contact.

### The decomposition rates of straws

The decomposition rates (*k*) of the straws with soil contact (AmbientS and UVBS) were higher than those without soil contact (Ambient and UVB) for maize straw but not for wheat straw (Table 2). In addition, compared with the Block condition of both straws, the decomposition rates (*k*) of Ambient and UVB for both straws were greater (*P* < 0.05, Table 2), indicating that light exposure promoted the decomposition rates of both straws.

### NMR spectra analysis

The ^13^C cross polarization/total sideband suppression (CP/TOSS) NMR spectra of the initial and decomposed wheat and maize straws are shown in Figures S1 and S2 (Supplementary information), respectively. The spectra of wheat and maize straws decomposed for 3 and 8 months were not included because they were similar to those of the initial straw samples and 12-month residues, respectively.

The spectra of the initial wheat (Figure S1(a)) and maize (Figure S2(a)) straw samples are quite similar, both showing strong signals from O-alkyl carbons, due to the presence of carbohydrates (Table 3). Figures S1(a) and S2(a) portray dominant OCH signals around 72 ppm, OCH_2_ resonances around 62 ppm, and OCO anomeric signals around 105 ppm, which are indicative of carbohydrates, although significant aromatic signals (between 110 and 142 ppm, attributed to aromatic or olefinic carbons) were not observed. In addition, small signals between 0 and 45 ppm indicated the presence of alkyl carbons. Moreover, weak signals for OCH_3_/NCH (around 56 ppm) and COO/N-C=O (around 173 ppm) were observed (Figures S1(a) and S2(a)); these signals were partially related to the amides of peptides/proteins. These results indicated that the dominant compounds in the initial wheat and maize straws were carbohydrates (cellulose and/or hemicellulose), whereas proteins, lipids and lignin were present in much smaller amounts.

Carbohydrate carbon signals continued to predominate in all spectra of the straw samples decomposed for 12 months (Figures S1 and S2). The spectra of 12-month decomposed wheat and maize samples showed stronger alkyl carbon signals ranging from 0 and 45 ppm, aromatic carbon resonances between 110–142 ppm, aromatic C-O carbon signals between 142–165 ppm, and COO/N-C=O carbon signals between 165–190 ppm relative to those of the initial samples, except for the spectrum of the Block maize straw (Figures S1 and S2). The spectrum of the Block maize straw (Figure S2(b)) showed smaller peaks for COO/N-C=O (around 173 ppm), aromatics (between 110–142 ppm) and CCH_2_ (around 33 ppm) relative to those of maize straw subject to light exposure without soil contact (Ambient and UVB) (Figure S2).

### The proportions of functional groups

Based on Table 3, wheat and maize straws showed increased alkyl C, and decreased O-alkyl and alkyl O-C-O after the 12-month decomposition period, except for the Block conditions of both the wheat and maize straws. The O-alkyl carbons of both the wheat and maize straws under the Block treatments were greater than those of Ambient and UVB, whereas the alkyl carbons showed the opposite trend. These results indicated that the O-alkyl carbons could have been associated with photochemically labile structural moieties, and the alkyl carbons (0–45 ppm) could have been photochemically inactive functional groups in straw.

### Alkyl/O-alkyl ratios and aromaticity

**Figure 2:**
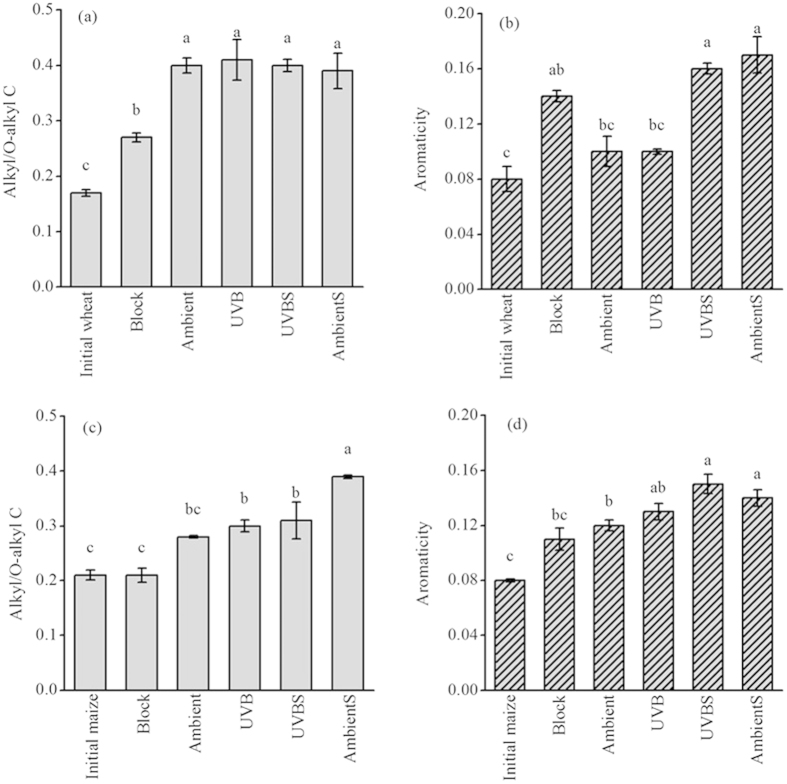
Alkyl/O-alkyl ratio and aromaticity of wheat (a,b) and maize (c,d) straws after 12 months of decomposition. Error bars indicate standard errors (n = 3). Lowercase letters denote significant differences between treatment groups at the 5% level according to Tukey’s test.

The ratios of alkyl/O-alkyl and aromaticity can be used as indexes of decomposition dynamics. The alkyl/O-alkyl ratios of the decomposed wheat straw were larger than those of the maize straw, except for AmbientS (Fig. 2). The alkyl/O-alkyl ratios of the wheat straw did not show significant differences between the four light exposure treatments, whereas UV-B significantly (*P* < 0.05) decreased the alkyl/O-alkyl ratio of maize straw in contact with soil. The alkyl/O-alkyl ratios of Ambient and UVB for both the wheat and maize straws were higher than those of Block (*P* < 0.05), except for that of maize straw under Ambient treatment, indicating that higher degrees of decomposition were obtained with light exposure. The aromaticity of wheat straw with soil contact was higher than that without soil contact. However, the aromaticity of maize straw did not show any difference between treatments with and without soil contact under elevated UV-B exposure. Light exposure did not play a significant role in aromaticity of wheat and maize straw without soil contact compared with those of Block (*P* > 0.05). Elevated UV-B levels did not significantly affect the aromaticity of either the wheat or maize straw, with or without soil contact (Fig. 2).

### Principal component analysis

**Figure 3:**
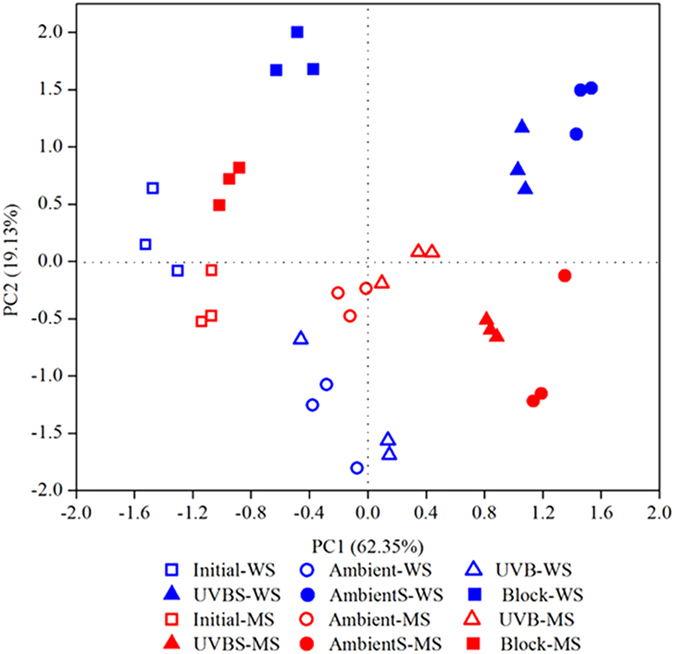
Principal component analysis of the compositions of the carbon functional groups. W = wheat straw, M = maize straw.

Figure 3 shows the results of a principal component analysis (PCA), which was carried out based on the relative proportion of the carbon functional groups. According to the results, the points plotted for the Block and the light exposure treatments without soil contact clearly fell into separate regions, indicating that light exposure affected the decomposition of the carbon functional groups (Fig. 3). In addition, both straw samples with soil contact were separated from those without soil contact (Fig. 3), indicating that soil contact played a role in decomposing the carbon functional groups. The chemical structure of both types of straw changed considerably over time, indicating that the structures of the initial and decomposed straw samples were distinctly different after 12 months.

## Discussion

According to our results, solar exposure is an important driver of the decomposition process of straw. Compared with the Block condition for both straws, exposure to 12-month UVB and Ambient increased the mass loss of wheat and maize straws due to photochemical mineralization (*P* < 0.05). These findings are consistent with previous reports, which have indicated that increased exposure to UV-B accelerates the decomposition rates of litter in arid and semi-arid ecosystems^9^,^10^,^14^. However, the mesic ecosystem considered in the present study differs from arid and semi-arid ecosystems with regard to straw decomposition. Austin and Vivanco^9^ showed that solar radiation was the most important driver of litter decomposition in a Patagonian steppe (a semi-arid ecosystem). In the present study, UV-B exposure contributed 14% of the mass loss for wheat straw and 19% of that for maize straw.

It should be noted that it would not be possible to evaluate the quantitative contribution of soil contact to straw mass loss due to the absence of the treatment of dark control with soil contact (BlockS). We speculate that the mass loss of a BlockS treatment was likely higher than the treatments with sunlight exposure without soil contact, since the microbial degradation appears to be a more important driver in straw decomposition than photodegradation under the mesic conditions^18^. In addition, a previous study investigated the difference of litter mass loss with total blocked radiation under conditions where microbial decomposition was either inhibited or not, and found that the alteration of biotic activity with total blocked radiation had no effect on litter decomposition^9^. Nevertheless, further studies are needed to quantitatively evaluate the respective contribution of UV-B radiation and soil contact to straw decomposition in mesic ecosystems.

Our results showed that UV-B radiation increased the decomposition rates of litter in light-exposed treatments compared with the no-light-exposure control, as supported by Brandt *et al.*^10^. A previous study found that shading significantly reduces the decomposition rates of litter in mixed-grass prairie^25^, indicating that light exposure plays an important role in litter decomposition. Our results demonstrated that both Ambient and UVB conditions accelerated the decomposition of wheat and maize straws compared with Block for both straws (Fig. 1, *P* < 0.05); this finding is in agreement with that of Kochy and Wilson^25^, who found that UV blocking reduced the litter mass loss from 29% to 17% in low-lignin litter.

During the process of straw decomposition, UV-B exposure can abiotically and biotically influence the decomposition process. The abiotic effect (or direct effect) is caused by direct photodegradation^12^,^26^, and the biotic effect (or indirect effect) is triggered by changes in the soil microbial community and the activity induced by UV-B exposure^27^,^28^,^29^,^30^. However, these two UV-B effects can work in opposite directions during decomposition, with increased UV-B exposure accelerating the rates of straw decomposition due to abiotic photochemical breakdown, whereas decreased biotic decomposition occurs as a result of reduced soil microbial processes^28^,^29^,^30^. Many studies have concluded that photodegradation is the primary controlling factor over litter decomposition because the soil microbial community and its activity are inhibited in arid or other harsh environments^9^,^10^,^14^. In mesic systems, biotic processes are the main factors in the determination of litter decomposition due to high microbial activity^18^. The effect of elevated UV-B exposure on the inhibition of the activity of microbial decomposers might overshadow the effect of photodegradation, resulting in a lower decomposition rate. Several studies have examined the effect of enhanced UV-B exposure on litter decomposition with the addition of soil^9^,^18^. However, Austin and Vivanco^9^ observed no interactive effect between soil addition and UV-B exposure on litter mass loss due to the absence or inhibition of microbial activity in moisture-limited environments; these results are inconsistent with those presented in this study, which revealed a significant interactive effect between UV-B exposure and soil contact (Table 1, *P* < 0.05).

Soil contact was another important driver in the straw decomposition process and clearly played a role in the regulation of straw photodegradation. Elevated 12-month UV-B exposure without soil (UVB) did not significantly affect the mass loss of the wheat and maize straws compared with Ambient. In addition, greater UV-B exposure with soil contact (UVBS) reduced the mass loss compared with AmbientS (*P* < 0.05, Fig. 1), probably due to the inhibition of soil microbial activity. However, this conjecture will need to be substantiated by further investigation. Therefore, our results provided evidence for our hypothesis that UV-B exposure influences straw decomposition rates depending on soil contact (microbial activity). Day *et al.*^14^ showed that twigs on the soil surface had lower decay rates than those placed on a balcony without soil contact in the desert, where microbial decomposition was inherently slow. Smith *et al.*^18^ found that the effects of UV-B exposure on mass loss under relatively wet conditions (4-day precipitation frequency) were different between the control soil and the reduced-microbial soil when biotic processes were the primary driver of decomposition, which was similar to our argument.

Elevated UV-B radiation changed the chemical properties of the straw during decomposition. UV-B radiation can directly mineralize organic materials and release C-based gases such as CH_4_ and CO_2_^11^. We found that elevated UV-B radiation decreased straw C concentrations in wheat straw without soil contact. In addition, elevated UV-B exposure increased the C:N ratios of both wheat and maize straws with soil contact, but not those without soil contact. Day *et al.*^14^ found that exposure to UV-B had no effect on the concentrations of litter C, N and C:N ratios. Our results showed that the N concentrations of both straws without soil contact were not affected by UV-B exposure, which was inconsistent with the results presented by Brandt *et al.*^10^, who demonstrated that elevated UV-B slowed N immobilization in litter by suppressing the microbial ability related to N immobilization.

The effects of UV-B radiation on the decomposition of chemical structures in straw have not been fully elucidated. Our study indicated that the chemical structure of straw was strongly influenced by soil contact and light exposure, as revealed by information recorded for carbon functional groups (Table 3). Alkyl carbons are considered to be photo-resistant compounds that resist photochemical degradation processes^31^. After the 12-month decomposition period, an increase in alkyl C region (0–45 ppm) was evident based on the wheat (Figure S1) and maize (Figure S2) straw spectra. The increase in alkyl C was possibly associated with either an increase in cross-linking of the long-chain alkyl compounds^32^ or the selective preservation of resistant aliphatic biomacromlecules^33^. The production of alkyl carbons via photodegradation have been supported by a series of studies^31^,^34^,^35^. Therefore, the photodegradation products of alkyl components may account for the greater alkyl C in the straw exposed to the UVB and Ambient conditions compared to the Block conditions of both straws.

The rapid mineralization of labile components, such as O-alkyl carbons, is the dominant process during early decomposition^36^. Compared with the treatments that lacked soil contact, the sharp decrease in the O-alkyl of AmbientS and UVBS of wheat straw (Table 3) may be attributed to the decomposition of labile carbohydrates^37^,^38^. Some studies have demonstrated that the cellulose and hemicellulose in straw are preferentially susceptible to microbial decomposition^12^,^14^ and UV degradation^10^. The increase in alkyl carbon with decomposition coincides with a decline in O-alkyl carbons^39^, and thus the alky/O-alkyl ratio has been chosen as an indicator for estimating the degree of decomposition in many studies. Lorenz *et al.*^40^ showed an increase in the alkyl/O-alkyl ratio for black spruce and Norway spruce in Germany after a 12-month decomposition period, which is in agreement with our results based on wheat straw degradation.

An increase in aromaticity was observed in both the wheat and maize straws during decomposition, except for the Ambient and UVB treatments of wheat straw and the Block of maize straw (Fig. 2). This result can be explained by the fact that the fraction of aromatic C significantly increased as decomposition proceeded. A previous study reported that leaf litter exposed to near-ambient UV-B had lower lignin compared with that of reduced UV-B treatment^14^. Similarly, Helms *et al.* (2014) found that photodegradation, especially that of aromatics, resulted in the greater mass loss of DOM under near-ambient UV-B exposure than reduced UV-B exposure^31^. During photodegradation, aromatic carbons, especially those attributed to lignin, are preferentially decomposed because lignin is an effective light-absorbing compound over a wide range of wavelengths^15^. However, our results indicated that exposure to solar radiation did not affect the relative proportions of aromatic C of straw, which is inconsistent with the results of studies indicating that lignin strongly absorbs UV and is prone to UV-B photodegradation^41^,^42^.

UV-B exposure significantly affected the decomposition of straw without soil contact in the mesic ecosystem via photodegradation compared with no sunlight radiation; however, the role of UV-B radiation in straw decomposition depended on soil contact. Our findings supported the hypothesis proposed in previous studies^43^,^44^, specifically, that the role of photodegradation depends on the balance between biotic and abiotic drivers in the decomposition process.

In summary, light exposure strongly decreased C:N ratios and increased decomposition rates of both straws compared with those of Block. Greater UV-B exposure resulted in increasing decomposition rates of both the wheat and maize straws without soil contact when compared with the Block samples but did not show a significant difference relative to Ambient. Elevated UV-B radiation decreased decomposition rates of both the wheat and maize straws with soil contact. Our results indicated that O-alkyl carbons decreased via photodegradation, whereas alkyl carbons increased. The effects of light exposure on carbon functional groups of the straws with soil contact were different from those without soil contact. Ultimately, our results highlighted light exposure as a potential driver of straw decomposition in mesic ecosystems and indicated that soil contact significantly regulated the effect of photodegradation on straw decomposition. Our study facilitated a better understanding of both the direct and indirect effects of photodegradation during the straw decomposition process.

## Methods

### Straw and soil sampling

Two types of straw residues (wheat (*Triticum aestivuml*) and maize (*Zea mays* L.) straw) and soil (0–20 cm layer) were acquired from Fengqiu Agro-ecological Experimental Station (35°00′ N, 114°24′ E), Chinese Academy of Sciences, Fengqiu County, Henan Province, China. The wheat and maize straw samples were collected in June and October 2012 after the harvests of winter wheat and summer maize, respectively. The straws were oven-dried at 65 °C and then cut into pieces measuring 2 cm in length. The mass loss of straw was measured by weighing the oven-dried (65 °C) samples. Cellulose, hemicelluloses and proteins were labile compounds, whereas lignin was a recalcitrant compound in both the wheat and maize straws. After the visible plant residues and stones were removed, the soils were air-dried and passed through a 2-mm sieve. The soil contained 6.8 g kg^−1^ organic matter and had a pH of 8.2, and its total N, P and K contents were 0.6, 0.5 and 18.4 g kg^−1^, respectively.

### Experimental setup

The straw decomposition experiment was conducted in a greenhouse at the Institute of Soil Science, Chinese Academy of Sciences, Nanjing, Jiangsu Province, China (32°07′ N, 118°78′ E) in December 2012. The greenhouse was maintained at 25 °C and 20% relative humidity. The mean annual temperature and precipitation in this area are 16.1 °C and 1053 mm, respectively.

The straw samples were placed in litterbags and then were subjected to three UV-B levels (ambient UV-B, elevated UV-B and no sunlight control) and two soil contact levels (with and without soil contact). The five experimental treatments were (1) litterbags placed on the bottom of glass containers without soil contact under ambient solar exposure (Ambient), (2) litterbags placed on the bottom of glass containers without soil contact under elevated UV-B exposure (UVB), (3) litterbags placed on the bottom of glass containers without either soil contact or sunlight (Block), (4) litterbags on the soil surface under ambient solar exposure (AmbientS) and (5) litterbags on the soil surface under elevated UV-B exposure (UVBS). A block control with soil contact was not included. Each treatment was conducted with three replicates. Ambient was exposure to ambient solar radiation. Aclar plastic film (Aclar Type 22A film, 125-μm thickness, DuPont Co., Beijing, China), which allowed the transmission of 95% of solar radiation, was used for the Ambient condition. The Block treatment was employed as a control; Mylar filters (Mylar-D, 125-μm thickness, DuPont Co., Beijing, China) covered with reflective aerosol paint were used to effectively block 90% of solar radiation. UVB was regulated using artificial irradiance from fluorescent UV-B lamps (UV-B313EL, Beijing Lighting Research Institute, Beijing, China) wrapped with cellulose Acetate film (0.07 mm, DuPont Co., Beijing, China) to remove all ultraviolet-C (UV-C). The films were replaced every month to maintain acceptable limits of light transmission. The fluorescent UV-B lamps were operated for 7 h daily from 09:00 to 16:00 and were regulated to maintain 15% enhancement above the ambient UV-B exposure. The UV-B energy was measured using a UV-297 radiometer (Photoelectric Instrument Factory of Beijing Normal University, Beijing, China). During straw decomposition, the mean elevated UV-B level was 56 μW cm^−2^. The litterbags were randomly placed in open-top glass containers (75 × 75 × 30 cm) with UV-B lamps positioned 30 cm above the bottom of the containers. For treatments with soil (AmbientS and UVBS), approximately 25 kg of sieved soil was added to the containers beneath the litterbags. To minimize the introduction of potential errors due to location differences within the containers, the litterbags were transferred to different positions every two weeks to ensure that each sample received the same amount of UV-B radiation.

### Straw decomposition and chemical analysis

The litterbag method was used to determine the rates of straw decomposition. Nylon fabric, which blocked 10.1% of the incoming solar radiation, was used. The mesh litterbags, which measured 15 cm long and 10 cm wide, were filled with 5 g of air-dried wheat or maize straw and pinned to the soil surface using pins (with soil contact) or placed on the bottom of the container without soil (without soil contact). The decomposition experiments lasted for 12 months, from December 2012 to December 2013. Three litterbags were randomly taken from each treatment after 3, 8 and 12 months, respectively. Upon removal from the containers, the soil was removed from the litterbags. The straw samples were oven-dried at 65 °C and weighed. The rate of straw decomposition was expressed as the percentage of mass loss of straw at different decomposition times. The following exponential model was used to express the behavior of straw decomposition:


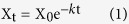


where X_t_ is the weight percentage of the mass remaining at time t (years), X_0_ is the weight percentage of the initial mass and *k* is the decomposition rate. The total C and N concentrations of the straw samples were determined by dichromate oxidation and Kjeldahl digestion, respectively^45^.

### ^13^C CP/TOSS NMR experiments

Information regarding the carbon functional groups of straw was obtained by solid-state ^13^C CP/TOSS using a Bruker Avance 400 spectrometer (Bruker BioSpin, Rheinstetten, Germany) at the 100.6 MHz frequency of ^13^C. The straw samples were first ground and then passed through a 0.074-mm sieve. Approximately 100 mg of straw sample was packed in a 4-mm rotor and then spun at a speed of 5 kHz. The contact time was 1 ms, and the recycle delays were 2 s. The number of scans was 2000 for each sample.

The spectra were integrated into eight chemical shift regions, with assignments as follows^46^,^47^: 0–45 ppm, nonpolar alkyl; 45–60 ppm, NCH/OCH_3_; 60–93 ppm, O–alkyl; 93–110 ppm, alkyl O–C–O; 110–142 ppm, aromatic C–C+/H; 142–165 ppm, aromatic C–O; 165–190 ppm, COO/N–C=O and 190–220 ppm, ketones or aldehydes. Though NMR spectra obtained with cross polarization techniques are semi-quantitative, they can still be used to analyze the structural alterations during the decomposition of straw samples. The indices for estimating the degree of decomposition, expressed as the ratios of alkyl C to O-alkyl C and aromaticity, were calculated according to the approach proposed by Baldock *et al.*^39^ and Wang *et al.*^37^.

### Statistical Analysis

All data are presented as the mean value ± standard error (SE) of three replicates. The significance of difference between the experimental treatments was detected using one-way analysis of variance (ANOVA), and Turkey’s test was used to compare the means at a significance level of *P* < 0.05. The effects of UV-B radiation (no-sunlight control, ambient UV-B radiation and elevated UV-B radiation) and soil contact (with and without soil contact) on mass loss were tested using two-way ANOVA analysis. Principal component analysis (PCA) of the functional group composition of each sample was performed to discriminate the differentiation in the chemical structure of straw residues. All analyses were carried out in the Statistical Software Package for Social Science (SPSS 17.0).

## Additional Information

**How to cite this article**: Zhou, G. *et al.* Mass loss and chemical structures of wheat and maize straws in response to ultraviolet-B radiation and soil contact. *Sci. Rep.* **5**, 14851; doi: 10.1038/srep14851 (2015).

## Supplementary Material

Supplementary Information

## Figures and Tables

**Table 1:** Effects of UV-B radiation and soil contact on the mass loss of straw after 12 months of decomposition.

Source of variance	Wheat straw mass loss			Maize straw mass loss		
	df	F	*P*	df	F	*P*
UV-B radiation (UVBR)	2	9.657	0.005	2	27.763	<0.001
Soil contact (SC)	1	51.998	<0.001	1	66.002	<0.001
UVBR × SC	1	17.761	0.002	1	6.831	0.026

Df, F and *P* refer to the degree of freedom, F-value and significance, respectively.

**Table 2:** Chemical properties and decomposition rates (*k*-value) calculated by the exponential decay model (X_t_ = X_0_e^−*k*t^) for wheat and maize straw after one year of decomposition.

Straw	Treatment	C concentration (g kg^−1^)	N concentration (g kg^−1^)	C:N ratio	Decomposition rate (year^−1^)
Wheat straw	Initial	419.51 ± 2.37a	6.71 ± 0.20a	62.63 ± 1.56d	
	Block	373.59 ± 0.88b	2.74 ± 0.05c	136.50 ± 2.98a	0.16 ± 0.00c
	Ambient	379.38 ± 2.86b	3.14 ± 0.01c	120.78 ± 0.93b	0.42 ± 0.11b
	UVB	350.63 ± 1.25c	3.04 ± 0.14c	115.65 ± 4.59b	0.51 ± 0.09b
	UVBS	303.33 ± 3.32d	3.82 ± 0.11b	79.66 ± 3.02c	0.64 ± 0.11b
	AmbientS	241.27 ± 1.01e	4.23 ± 0.08b	57.12 ± 1.17d	1.04 ± 0.08a
Maize straw	Initial	438.57 ± 16.87a	6.15 ± 0.82a	73.62 ± 8.44de	
	Block	401.03 ± 2.24b	2.81 ± 0.02c	142.91 ± 1.70a	0.20 ± 0.05d
	Ambient	348.54 ± 1.29c	2.95 ± 0.02bc	118.22 ± 0.93b	0.45 ± 0.04c
	UVB	328.04 ± 4.13cd	3.25 ± 0.13bc	101.15 ± 3.28bc	0.51 ± 0.02c
	UVBS	301.87 ± 0.77de	3.35 ± 0.09bc	90.10 ± 1.75cd	0.75 ± 0.04b
	AmbientS	280.71 ± 1.58e	4.54 ± 0.15ab	61.99 ± 2.23e	1.02 ± 0.05a

All values are presented as mean ± standard error (n = 3). The lowercase letters within each column denote significant differences between the treatments for wheat or maize straw at the *P* < 5% level according to Tukey’s test.

**Table 3:** The assignment of functional groups at different chemical shift regions and their relative proportions in the total spectral area determined by ^13^C CP/TOSS.

Group	190–220 ppm	165–190 ppm	142–165 ppm	110–142 ppm	93–110 ppm	60–93 ppm	45–60 ppm	0–45 ppm
assignment	ketones/aldehydes	COO/N–C=O	aromatic C–O	aromatic C–C+/H	alkyl O–C–O	O–alkyl	NCH/OCH_3_	alkyl
Wheat straw								
Initial	0.5 ± 0.4b	3.6 ± 0.8c	2.0 ± 0.5bc	5.6 ± 0.3b	13.0 ± 0.1a	57.5 ± 1.1a	6.0 ± 0.2c	11.8 ± 0.4b
Block	1.9 ± 0.2a	4.1 ± 0.2bc	3.5 ± 0.1ab	6.3 ± 0.3b	10.5 ± 0.1b	53.8 ± 0.2a	7.4 ± 0.3abc	12.5 ± 0.5b
Ambient	1.2 ± 0.3ab	5.0 ± 0.4abc	2.0 ± 0.3bc	6.6 ± 0.7b	10.0 ± 0.1bc	45.7 ± 0.9b	7.3 ± 0.5bc	22.2 ± 0.6a
UVB	1.0 ± 0.2ab	4.1 ± 0.4bc	2.0 ± 0.1c	6.7 ± 0.2b	10.1 ± 0.3bc	45.4 ± 1.4b	8.1 ± 0.1ab	22.6 ± 1.4a
UVBS	1.8 ± 0.2ab	5.9 ± 0.1ab	3.8 ± 0.2a	9.3 ± 0.3a	9.8 ± 0.2bc	41.1 ± 0.2c	8.1 ± 0.3ab	20.2 ± 0.5a
AmbientS	1.5 ± 0.4ab	6.6 ± 0.2a	4.3 ± 0.5a	10.1 ± 0.6a	9.4 ± 0.4c	39.7 ± 0.2c	9.1 ± 0.6a	19.3 ± 1.4a
Maize straw								
Initial	0.8 ± 0.1a	3.8 ± 0.5d	1.7 ± 0.2b	5.6 ± 0.2c	12.0 ± 0.3a	54.9 ± 0.8a	7.3 ± 0.2cd	13.9 ± 0.4c
Block	0.9 ± 0.0a	4.0 ± 0.3cd	2.7 ± 0.2a	6.8 ± 0.5bc	12.0 ± 0.1a	52.9 ± 0.1a	7.0 ± 0.5d	13.7 ± 0.8c
Ambient	0.8 ± 0.2a	5.2 ± 0.2bcd	2.9 ± 0.2a	7.1 ± 0.2abc	10.8 ± 0.1b	48.4 ± 0.2b	8.4 ± 0.1bcd	16.4 ± 0.1b
UVB	1.2 ± 0.4a	5.3 ± 0.4abc	3.1 ± 0.3a	8.3 ± 0.2ab	10.7 ± 0.1b	45.7 ± 0.2c	8.8 ± 0.5bc	16.9 ± 0.6b
UVBS	1.2 ± 0.2a	5.6 ± 0.1ab	3.6 ± 0.1a	8.8 ± 0.5a	10.4 ± 0.4b	44.3 ± 1.0c	9.3 ± 0.5ab	16.8 ± 1.4b
AmbientS	0.9 ± 0.3a	6.7 ± 0.1a	3.5 ± 0.2a	8.2 ± 0.3ab	9.2 ± 0.1c	41.2 ± 0.1d	10.7 ± 0.2a	19.6 ± 0.2a

The lowercase letters within each column denote significant differences between the treatments at the *P* < 5% level according to Tukey’s test.

## References

[b1] Tian G. *et al.* Effects of residue quality and climate on plant residue decomposition and nutrient release along the transect from humid forest to Sahel of West Africa. Biogeochemistry 86, 217–229 (2007).

[b2] Grace J. & Rayment M. Respiration in the balance. Nature 404, 819–820 (2000). 10786772 10.1038/35009170

[b3] Janzen H. H. Carbon cycling in earth systems-a soil science perspective. Agr. Ecosyst. Environ. 104, 399–417 (2004).

[b4] King J. Y., Brandt L. A. & Adair E. C. Shedding light on plant litter decomposition: advances, implications and new directions in understanding the role of photodegradation. Biogeochemistry 111, 57–81 (2012).

[b5] Henry H. A. L., Brizgys K. & Field C. B. Litter decomposition in a california annual grassland: Interactions between photodegradation and litter layer thickness. Ecosystems 11, 545–554 (2008).

[b6] Song X. Z. *et al.* Interactive effects of elevated UV-B radiation and N deposition on decomposition of Moso bamboo litter. Soil Biol. Biochem. 69, 11–16 (2014).

[b7] Messenger D. J., Fry S. C., Yamulki S. & McLeod A. R. Effects of UV-B filtration on the chemistry and decomposition of *Fraxinus excelsior* leaves. Soil Biol. Biochem. 47, 133–141 (2012).

[b8] Newsham K. K. *et al.* Negligible influence of elevated UV-B radiation on leaf litter quality of *Quercus robur*. Soil Biol. Biochem. 33, 659–665 (2001).

[b9] Austin A. T. & Vivanco L. Plant litter decomposition in a semi-arid ecosystem controlled by photodegradation. Nature 442, 555–558 (2006). 16885982 10.1038/nature05038

[b10] Brandt L. A., King J. Y. & Milchunas D. G. Effects of ultraviolet radiation on litter decomposition depend on precipitation and litter chemistry in a shortgrass steppe ecosystem. Global Change Biol. 13, 2193–2205 (2007).

[b11] Brandt L. A., Bohnet C. & King J. Y. Photochemically induced carbon dioxide production as a mechanism for carbon loss from plant litter in arid ecosystems. J. Geophys. Res. Biogeosci (2005–2012). 114, G2 (2009).

[b12] Rozema J. *et al.* Stratospheric ozone reduction and ecosystem processes: Enhanced UV-B radiation affects chemical quality and decomposition of leaves of the dune grassland species *Calamagrostis epigeios*. Plant Ecol. 128, 284–294 (1997).

[b13] Brandt L. A., King J. Y., Hobbie S. E., Milchunas D. G. & Sinsabaugh R. L. The role of photodegradation in surface litter decomposition across a grassland ecosystem precipitation gradient. Ecosystems 13, 765–781 (2010).

[b14] Day T. A., Zhang E. T. & Ruhland C. T. Exposure to solar UV-B radiation accelerates mass and lignin loss of *Larrea tridentata* litter in the Sonoran Desert. Plant Ecol. 193, 185–194 (2007).

[b15] Austin A. T. & Ballare C. L. Dual role of lignin in plant litter decomposition in terrestrial ecosystems. Proc. Natl. Acad. Sci. 107, 4618–4622 (2010). 20176940 10.1073/pnas.0909396107 PMC2842047

[b16] Anesio A. M., Tranvik L. J. & Graneli W. Production of inorganic carbon from aquatic macrophytes by solar radiation. Ecology 80, 1852–1859 (1999).

[b17] Gallo M. E., Sinsabaugh R. L. & Cabaniss S. E. The role of ultraviolet radiation in litter decomposition in and ecosystems. Appl. Soil Ecol. 34, 82–91 (2006).

[b18] Smith W. K., Gao W., Steltzer H., Wallenstein M. D. & Tree R. Moisture availability influences the effect of ultraviolet-B radiation on leaf litter decomposition. Global Change Biol. 16, 484–495 (2010).

[b19] Uselman S. M., Snyder K. A., Blank R. R. & Jones T. J. UVB exposure does not accelerate rates of litter decomposition in a semi-arid riparian ecosystem. Soil Biol. Biochem. 43, 1254–1265 (2011).

[b20] Verhoef H. A., Verspagen J. M. H. & Zoomer H. R. Direct and indirect effects of ultraviolet-B radiation on soil biota, decomposition and nutrient fluxes in dune grassland soil systems. Biol. Fert. Soils 31, 366–371 (2000).

[b21] Johnson D. Response of terrestrial microorganisms to ultraviolet-B radiation in ecosystems. Res. Microbiol. 154, 315–320 (2003). 12837506 10.1016/S0923-2508(03)00078-0

[b22] Barnes P. W., Throop H. L., Hewins D. B., Abbene M. L. & Archer S. R. Soil coverage reduces photodegradation and promotes the development of soil-microbial films on dryland leaf litter. Ecosystems 15, 311–321 (2012).

[b23] Holland E. A. & Coleman D. C. Litter placement effects on microbial and organic-matter dynamics in an agroecosystem. Ecology 68, 425–433 (1987).

[b24] Thurow T. L. Decomposition of grasses and forbs in coastal savanna of southern Somalia. Afr. J. Ecol. 27, 201–206 (1989).

[b25] Kochy M. & Wilson S. D. Litter decomposition and nitrogen dynamics in aspen forest and mixed-grass prairie. Ecology 78, 732–739 (1997).

[b26] Gehrke C., Johanson U., Callaghan T. V., Chadwick D. & Robinson C. H. The impact of enhanced ultraviolet-B radiation on litter quality and decomposition processes in Vaccinium leaves from the subarctic. Oikos 72, 213–222 (1995).

[b27] Moody S. A., Newsham K. K., Ayres P. G. & Paul N. D. Variation in the responses of litter and phylloplane fungi to UV-B radiation (290–315 nm). Mycol. Res. 103, 1469–1477 (1999).

[b28] Duguay K. J. & Klironomos J. N. Direct and indirect effects of enhanced UV-B radiation on the decomposing and competitive abilities of saprobic fungi. Appl. Soil Ecol. 14, 157–164 (2000).

[b29] Pancotto V. A. *et al.* Solar UV-B decreases decomposition in herbaceous plant litter in Tierra del Fuego, Argentina: potential role of an altered decomposer community. Global Change Biol. 9, 1465–1474 (2003).

[b30] Robson T. M. *et al.* Reduction of solar UV-B mediates changes in the Sphagnum capitulum microenvironment and the peatland microfungal community. Oecologia 140, 480–490 (2004). 15179579 10.1007/s00442-004-1600-9

[b31] Helms J. R. *et al.* Loss of optical and molecular indicators of terrigenous dissolved organic matter during long-term photobleaching. Aquat. Sci. 76, 353–373 (2014).

[b32] Kogelknabner I., Deleeuw J. W. & Hatcher P. G. Nature and distribution of alkyl carbon in forest soil profiles-implications for the origin and humification of aliphatic biomacromolecules. Sci. Total Environ. 118, 175–185 (1992).

[b33] Winkler A., Haumaier L. & Zech W. Insoluble alkyl carbon components in soils derive mainly from cutin and suberin. Org. Geochem. 36, 519–529 (2005).

[b34] Stubbins A. *et al.* Relating carbon monoxide photoproduction to dissolved organic matter functionality. Environ. Sci. Technol. 42, 3271–3276 (2008). 18522105 10.1021/es703014q

[b35] Stubbins A. *et al.* Illuminated darkness: Molecular signatures of Congo River dissolved organic matter and its photochemical alteration as revealed by ultrahigh precision mass spectrometry. Limnol. Oceanogr. 55, 1467–1477 (2010).

[b36] Zech W. *et al.* Factors controlling humification and mineralization of soil organic matter in the tropics. Geoderma 79, 117–161 (1997).

[b37] Wang X. Y., Sun B., Mao J. D., Sui Y. Y. & Cao X. Y. Structural convergence of maize and wheat straw during two-year decomposition under different climate conditions. Environ. Sci. Technol. 46, 7159–7165 (2012). 22668203 10.1021/es300522x

[b38] Wang W. J., Baldocka J. A., Dalala R. C. & Moody P. W. Decomposition dynamics of plant materials in relation to nitrogen availability and biochemistry determined by NMR and wet-chemical analysis. Soil Biol. Biochem. 36, 2045–2058 (2004).

[b39] Baldock J. A. *et al.* Assessing the extent of decomposition of natural organic materials using solid-state C-13 NMR spectroscopy. Aust. J. Soil Res. 35, 1061–1083 (1997).

[b40] Lorenz K., Preston C. M., Raspe S., Morrison I. K. & Feger K. H. Litter decomposition and humus characteristics in Canadian and German spruce ecosystems: information from tannin analysis and ^13^C CPMAS NMR. Soil Biol. Biochem. 32, 779–792 (2000).

[b41] Yue M., Li Y. & Wang X. L. Effects of enhanced ultraviolet-B radiation on plant nutrients and decomposition of spring wheat under field conditions. Environ. Exp. Bot. 40, 187–196 (1998).

[b42] Preston C. M., Bhatti J. S., Flanagan L. B. & Norris C. Stocks, chemistry, and sensitivity to climate change of dead organic matter along the Canadian boreal forest transect case study. Climatic Change 74, 223–251 (2006).

[b43] Pancotto V. A., Sala O. E., Robson T. M., Caldwell M. M. & Scopel A. L. Direct and indirect effects of solar ultraviolet-B radiation on long-term decomposition. Global Change Biol. 11, 1982–1989 (2005).

[b44] Zepp R. G., Erickson D. J. 3rd, Paul N. D. & Sulzberger B. Interactive effects of solar UV radiation and climate change on biogeochemical cycling. Photochem. Photobiol. Sci. 6, 286–300 (2007). 17344963 10.1039/b700021a

[b45] Novozamsky I., Houba V. J. G., Vaneck R. & Vanvark W. A novel digestion technique for multi-element plant analysis. Commun. Soil Sci. Plan 14, 239–248 (1983).

[b46] Mao J. D. *et al.* Quantitative characterization of humic substances by solid-state carbon-13 nuclear magnetic resonance. Soil Sci. Soc. Am. J. 64, 873–884 (2000).

[b47] Mao J. D., Olk D. C., Fang X. W., He Z. Q. & Schmidt-Rohr K. Influence of animal manure application on the chemical structures of soil organic matter as investigated by advanced solid-state NMR and FT-IR spectroscopy. Geoderma 146, 353–362 (2008).

