# Supplementary material for: Mass loss and chemical structures of wheat and maize straws in response to ultraviolet-B radiation and soil contact
Source: Sci Rep. 2015 Oct 1;5:14851. doi: 10.1038/srep14851 (PMC4589766; doi:10.1038/srep14851)
Supplement: Supplementary Information [file srep14851-s1.pdf]

Supplementary Information

Mass loss and chemical structures of wheat and maize straws in response to ultraviolet-B radiation and soil contact

Guixiang Zhou[1,2,3], Jiabao Zhang[1*], Jingdong Mao[4], Congzhi Zhang[1], Lin Chen[5], Xiuli Xin[1], Bingzi Zhao[1]

[1]State Key Laboratory of Soil and Sustainable Agriculture, Institute of Soil Science, Chinese Academy of Sciences, Nanjing 210008, China

[2] Jiujiang University, Jiujiang 332005, China

[3]University of Chinese Academy of Sciences, Beijing 100049, China

[4]Department of Chemistry and Biochemistry, Old Dominion University, 4541 Hampton Boulevard, Norfolk, Virginia 23529, United States

[5]Institute of Soil and Water Resources and Environmental Science, College of Environmental & Resource Sciences, Zhejiang University, Hangzhou 310058, China

Correspondence and requests for materials should be addressed to J.-B. Zhang. (email: jbzhang@issas.ac.cn).

Figure S1. ^{13}C CP/TOSS spectra of the initial and one-year decomposed wheat straw.

Figure S2. ^{13}C CP/TOSS spectra of the initial and one-year decomposed maize straw.

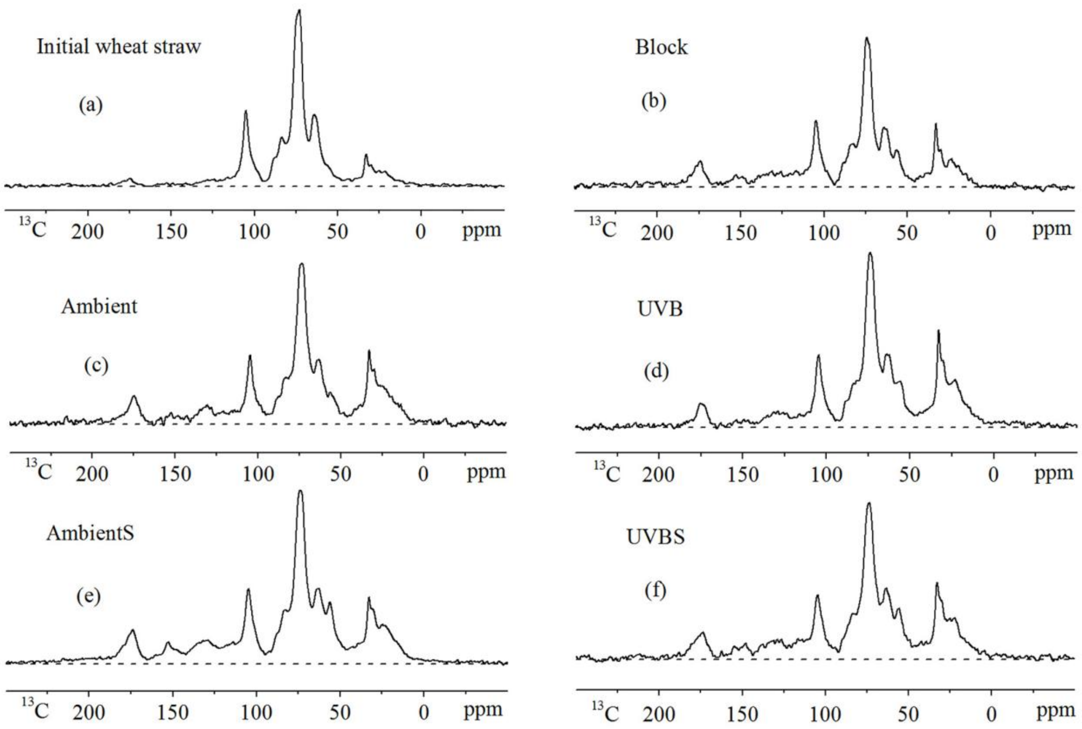

Figure S1. ^{13}C CP/TOSS spectra of the initial and one-year decomposed wheat straw.

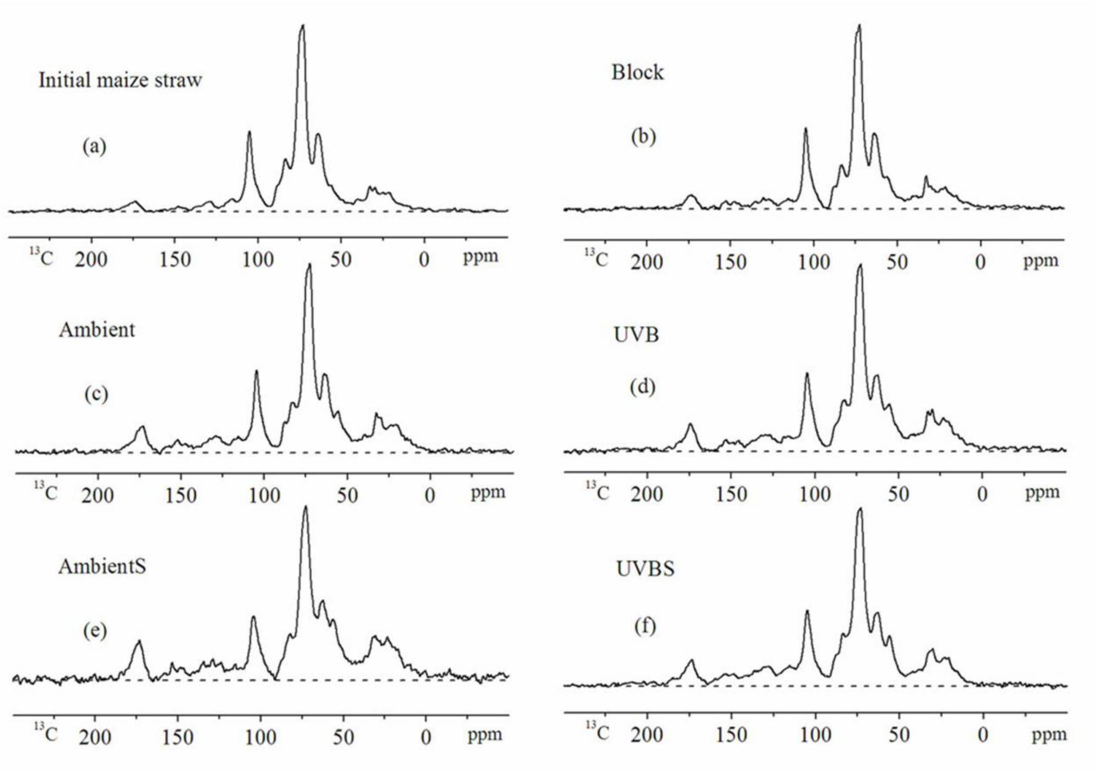

Figure S2. ^{13}C CP/TOSS spectra of the initial and one-year decomposed maize straw.
